# The Metric System

**Published:** 1907-03-23

**Authors:** 


### The Hospital

A Journal of

The Medical Sciences and Hospital Administration.

Vol. XLI.—No. 1071. SATURDAY, MARCH 23, 1907.

### The Metric System.

The movement in favour of the introduction of the metric system into the commerce and practice of the British Empire has recently assumed a somewhat active phase. Through the efforts of the Decimal Association the question has for a long time been kept more or less in evidence, and the subject of Imperial weights and measures appears on the official programme of the forthcoming Colonial Conference. Another evidence of activity on the part of the advocates of a decimal system of weights and measures is the introduction into the House of Commons, by Mr. B. S. Strauss, of a Bill proposing that from April 1, 1910, the standard kilogram and standard metre shall, respectively, be imperial standards of weight and measure for the purposes of the Weights and Measures Acts. In the commercial world, again, the metric system has achieved a distinct triumph in the announcement of a large manufacturing firm that in future the decimal weights and measures will be employed in all their business transactions. And, to come, perhaps, more closely to the immediate interests of our readers, it may be noted that the Therapeutical Society, at a recent meeting, and on the proposal of Dr. Geo. C. Crichton, discussed the introduction of the metric system into the British Pharmacopœia; and also as the standard to be used in the construction of prescriptions and the dispensing of medicines.

From all this, however, it must not be concluded that the metric system is one to which an unqualified welcome is afforded. If it can boast a special association proud to proclaim its merits, it has also to recognise the existence of an organisation arranged to play the part of *advocatus diaboli*. That the opposition is not without influence may be seen from the vote taken a few days ago at the meeting of the Associated Chambers of Commerce, where the resolution in favour of the compulsory adoption of the metric system managed to secure only a very narrow majority. Further, apart from opposition on principle, there are not a few critics who, as candid friends, point out some of the difficulties and risks which the decimal standard involves. Even in business transactions, it is urged, comparatively little advantage would accrue, unless, in addition to weights and measures, the coinage also was arranged on a decimal basis; and to this latter suggestion financial authorities would have much to say. Again, it is pointed out that the many-syllabled terms of the metric system, such, for example, as millimetre, kilogram, and the rest, are both cumbersome and inconvenient, and that they compare very badly in these respects with such plain and direct monosyllables as yard, pint, inch, etc. Some attempt has been made, it is true, to meet this difficulty by the introduction of simpler terms for the metric equivalents, but the abbreviated names have this drawback, that they fail to proclaim their arithmetical relationship to the allied metric terms, and thus one of the advantages of the decimal plan is lost. In connection with prescribing and dispensing the critics of the metric system have also pointed out a special risk, namely, that displacement of a decimal point is an error easily accomplished, and that it may carry serious consequences when it affects the quantities of medicines ordered for internal administration.

The question of the metric system and the controversies which are associated with it, will, apart from legislation, assume a practical aspect for the medical profession when the next edition of the Pharmacopœia comes under discussion. In the last issue metric weights and measures were used as alternatives to the imperial standards in the description of the composition of the official preparations, but the doses of the various remedies were stated in imperial equivalents only. If this arrangement was a compromise between two contending schools it was surely a singularly futile one. The key of the whole position, so far as medicine and pharmacy are concerned, is that physicians should write their prescriptions in metric terms, and this they will never do unless as students they learn to think of the doses of remedies in these terms. Hence, if the metric system is to be desired in medical practice, it must be adopted in stating the official doses. The Pharmacopœia Committee must make up its mind on the general question, and then either definitely adopt or definitely exclude the metric system. The present arrangement is neither logical nor convenient.

